# P-665. National Patterns in Use of Multiplex Respiratory Panels and Single Virus Testing for Patients with Community-Acquired Pneumonia

**DOI:** 10.1093/ofid/ofaf695.878

**Published:** 2026-01-11

**Authors:** Meggie Griffin, Valerie M Vaughn, Michael Pulia

**Affiliations:** University of Wisconsin-Madison School of Medicine and Public Health, Madison, Wisconsin; University of Utah, Salt Lake City, Utah; University of Wisconsin-Madison School of Medicine and Public Health, Madison, Wisconsin

## Abstract

**Background:**

Community-acquired pneumonia (CAP) is the leading infectious cause of death in the US and the etiology is often unknown. While COVID-19 and influenza testing can influence CAP treatment, multiplex panels are expensive and have limited evidence that they impact antimicrobial treatment or outcomes. We aimed to characterize longitudinal respiratory viral testing patterns for hospitalized and emergency department (ED) patients with CAP with emphasis on changes that occurred after the emergence of COVID-19.

**Figure 1.**
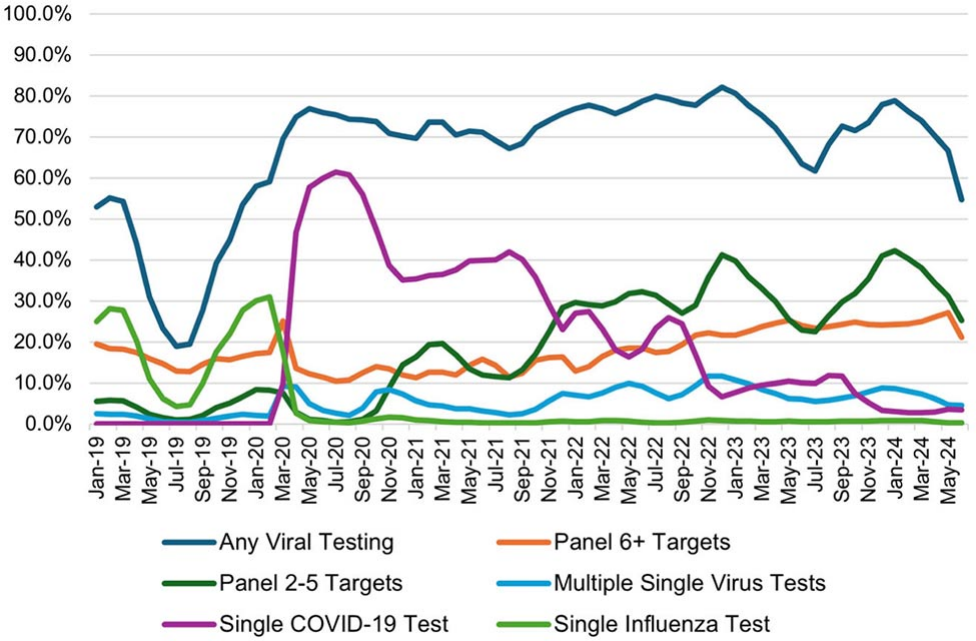
Monthly percentage of patients with community-acquired pneumonia that received respiratory viral testing by testing type (n = 4,826,353)

**Methods:**

We identified adult and pediatric CAP encounters including ED discharges, observations, and admissions by ICD-10 diagnosis codes in the PINC AI™ Healthcare Database (PHD) from January 2019 – June 2024. We excluded elective admissions, newborns, and encounters from hospitals reporting data for < 90% of study months. We classified respiratory viral testing into mutually exclusive, hierarchical categories: 1) panel with 6 or more targets (e.g., © Biofire testing), 2) panel with 2-5 targets, 3) multiple individual virus tests, 4) single individual virus test only (i.e., influenza A/B, COVID-19, or respiratory syncytial virus (RSV)). We calculated the monthly percentage of CAP encounters that received testing in each category and overall.

**Results:**

Of 4,826,353 eligible encounters for CAP from 615 hospitals in the PHD, 67.8% (3,274,028) underwent any respiratory viral testing. Patients with CAP had respiratory viral testing in 44.0% of encounters pre-COVID-19 and 73.3% post-COVID-19 emergence (Figure 1). Seasonal patterns, i.e. lower testing during summer, returned in 2023-24 but not to the lows seen in 2019. Single influenza testing dropped to nearly 0% and panels with 6+ targets showed a moderate rise in the post-COVID-19 period. Single COVID-19 testing was the most common testing type April 2020 – November 2021 after which panels with 2-5 targets became the most common.

**Conclusion:**

Respiratory viral testing for patients with CAP is higher following the COVID-19 pandemic with multiplex panels now the predominant respiratory viral test. Given the costs of expanded respiratory panels, future trials should investigate whether antibiotic treatment is necessary for viral CAP and thus whether such testing adds clinical value.

**Disclosures:**

All Authors: No reported disclosures

